# Effectiveness of nirsevimab immunization against RSV infection in preterm infants: a systematic review and meta-analysis

**DOI:** 10.3389/fimmu.2025.1581970

**Published:** 2025-04-17

**Authors:** Xiaopeng Wang, Li Kong, Xueou Liu, Panpan Wu, Lulu Zhang, Fangrui Ding

**Affiliations:** ^1^ Department of Neonatology, Tianjin Central Hospital of Obstetrics and Gynecology, Tianjin, China; ^2^ Tianjin Key Laboratory of Human Development and Reproductive Regulation, Tianjin, China; ^3^ Department of Neonatology, Nankai University Maternity Hospital, Tianjin, China; ^4^ Research Institute of Obstetrics and Gynecology, Tianjin Central Hospital of Obstetrics and Gynecology, Tianjin, China

**Keywords:** preterm (birth), RSV (respiratory syncytial virus), nirsevimab, infection, newborn

## Abstract

**Background:**

Respiratory Syncytial Virus (RSV) is one of the primary pathogen responsible for severe lower respiratory tract infections in preterm infants, placing a significant burden on patients, their families, and society. Nirsevimab, a recently developed RSV monoclonal antibody, has demonstrated promising efficacy in this population according to preliminary studies. However, there remains a need for comprehensive systematic reviews and meta-analyses to evaluate the effectiveness of nirsevimab in preventing RSV-related lower respiratory tract infections in preterm infants.

**Methods:**

A search of the PubMed and EMBASE databases was conducted to identify randomized controlled trials (RCTs) and observational studies assessing the prevention of RSV infection in preterm infants using nirsevimab. Relevant data were extracted and subjected to meta-analysis.

**Results:**

Five studies involving a total of 7,347 preterm infants (3,987 in the nirsevimab group and 3,360 in the control group) were included. The meta-analysis revealed that nirsevimab significantly reduced the incidence of medically attended RSV-associated lower respiratory tract infections (OR = 0.25; 95% CI: 0.15, 0.40; P < 0.0001) and hospitalizations due to RSV-associated lower respiratory tract infections (OR = 0.27; 95% CI: 0.19, 0.38; P < 0.0001).

**Conclusion:**

Nirsevimab significantly decreases the risk of RSV-related infection in preterm infants and represents a valuable intervention for RSV prevention in this vulnerable population.

**Systematic review registration:**

https://www.crd.york.ac.uk/prospero/, identifier CRD42025629937.

## Highlights

- Present study has addressed the effectiveness of nirsevimab immunization against RSV infection in the special cohort, namely: preterm infants.
- The present study suggest that nirsevimab could decrease the risk of RSV infection and hospitalization among preterm infants.
- The present study could encourage preterm infants to be immunized against RSV infection.

## 1 Introduction

Respiratory Syncytial Virus (RSV) is a leading cause of lower respiratory tract infections (LRTIs) in children under the age of five globally (1–3). Annually, RSV causes about 33 million LRTI cases, including 3.6 million hospitalizations and 26,300 deaths (1). RSV can lead to airway obstruction, cellular apoptosis, bronchitis, pneumonia, and respiratory failure requiring intensive care, particularly in infants under six months (4–8). Additionally, RSV infection increases the risk of long-term conditions like wheezing and asthma (9–11). The health risks, economic burdens on families, and strain on healthcare systems during RSV outbreaks highlight the urgent need for effective prevention and treatment strategies (1–3, 12, 13).

In the RSV-infected population, preterm infants have a significantly higher incidence and mortality rate of RSV-related LRTIs compared to full term groups. It was due to preterm infants lack of sufficient maternal antibodies and an underdeveloped immune system (14–17). Additionally, this group is more prone to developing severe illness after infection and has a higher risk of long-term complications (18, 19). Therefore, it is urgent to implement effective preventive measures for individuals with a history of preterm. Currently, there is no specific treatment for the RSV, the main treatment for RSV infection is supportive care such as oxygen therapy, mechanical ventilation, and symptomatic support treatments (2, 20). Thus, prevention becomes crucial in dealing with RSV infections.

The successful development of the nirsevimab monoclonal antibody has significantly benefited the prevention of RSV infection, particularly in high-risk populations such as preterm infants (21–26). This monoclonal antibody specifically targets the fusion protein (F protein) of RSV and inhibits the viral binding to host cells (21, 27–30). Recent studies have demonstrated its safety and efficacy, including in high-risk groups like preterm infants (21–24, 27–33). Currently, there are several studies designed to systematically evaluate the efficacy and provide early estimates on the effectiveness of nirservimab in infants (34–36). However, a comprehensive systematic review and meta-analysis focusing solely on preterm infants to evaluate the role of nirsevimab in preventing RSV-related infections is still lacking. Consequently, present study is to assess the efficacy of nirsevimab in preventing RSV-related infection in preterm infants by synthesizing data from existing studies. This study aims to provide robust scientific evidence for the use of nirsevimab in preterm infants, reducing the global RSV infection burden and improving their health.

## 2 Methods

This systematic review and meta-analysis, evaluating the efficacy of nirsevimab immunization in preterm infants, has been registered with PROSPERO 2021 (CRD42025629937) and reported in accordance with PRISMA guidelines (37).

### 2.1 Search strategy and selection criteria

To identify eligible studies, we conducted a comprehensive search of PubMed and Embase from their inception to November 4 2024. We updated our search March 28, 2025. Given the limited number of references related to nirsevimab, we included all references obtained using “nirsevimab” as a keyword, which yielded a reference list for further selection. We selected studies that reported outcomes regarding RSV-related LRTI in preterm infants who received nirsevimab immunization. Studies were initially screened based on their titles, abstracts, or full texts. The primary outcomes of interest were medically attended RSV-associated LRTI and hospitalization due to RSV-associated LRTI. The following studies were excluded (1): those not relevant to the meta-analysis topic (irrelevant to the subject) (2); those that did not report any of the primary outcomes (unsuitable outcomes) (3); reviews, editorials, conference papers, case reports, or animal studies (unsuitable study design) (4); those lacking a control group, specifically a non-immunized group(unsuitable population); and (5) those where the data for preterm infants could not be separated from the overall study population(unsuitable population). Two independent reviewers screened the studies according to the aforementioned criteria, and any discrepancies were resolved through consensus or by consulting a third reviewer.

### 2.2 Data extraction

The information encompassed general details (such as author, year, sample size, study setting, study design, observation period post-immunization with nirsevimab or placebo, etc.), participant characteristics (including gestational age), intervention specifics (type of monoclonal antibody, dosage, and administration route), and outcomes. Data extraction was performed independently by two reviewers according to the aforementioned criteria, and any discrepancies were resolved through consensus or with the involvement of a third reviewer.

### 2.3 Outcomes

Our study concentrated on primary outcomes, specifically medically attended RSV-associated LRTIs and hospitalizations due to RSV-associated LRTIs. Additional critical metrics included the incidence and duration of intensive care unit (ICU) admissions, the frequency and duration of supplemental oxygen administration, and the rate and duration of mechanical ventilation (MV) utilization. Medically attended RSV-related LRTIs or hospitalizations for RSV-associated LRTIs were defined as cases where participants received medical attention or were hospitalized with laboratory-confirmed RSV infections. RSV infection was confirmed through a positive polymerase chain reaction (PCR) test result.

### 2.4 Risk of bias assessment

The risk of bias assessment for each study was independently conducted by two reviewers utilizing the revised Cochrane risk of bias tool (38). This tool evaluates the following domains: bias arising from the randomization process, deviations from the intended interventions, missing data, outcome measurement, selection of reported results, and overall bias.

### 2.5 Statistical analysis

We conducted a meta-analysis to synthesize data from various studies. All outcomes were dichotomous, and we computed summary odds ratios and mean differences along with their 95% confidence intervals. We employed random-effects or fixed-effect models to aggregate the rates and adjusted estimates across studies, depending on the degree of heterogeneity (I²) between the estimates. Specifically, fixed-effect models were utilized when I² ≤ 50%, indicating low-to-moderate heterogeneity, while random-effects models were applied when I² ≥ 50%, signifying substantial heterogeneity. The I² values were interpreted as follows: 0–25% for no significant heterogeneity, 26–50% for low heterogeneity, 51–75% for moderate heterogeneity, and >75% for high heterogeneity. To assess the impact of each individual study on the pooled estimates, a sensitivity analysis was conducted employing the leave-one-out method. Publication bias was initially assessed through the visual analysis of the asymmetry of funnel plots, and was not assessed by application of Egger’s test due to the number of studies. Statistical analyses were conducted using the Cochrane Collaboration’s Review Manager software (RevMan 5.4).

## 3 Results

### 3.1 Search results and basic characteristics

**Figure 1:**
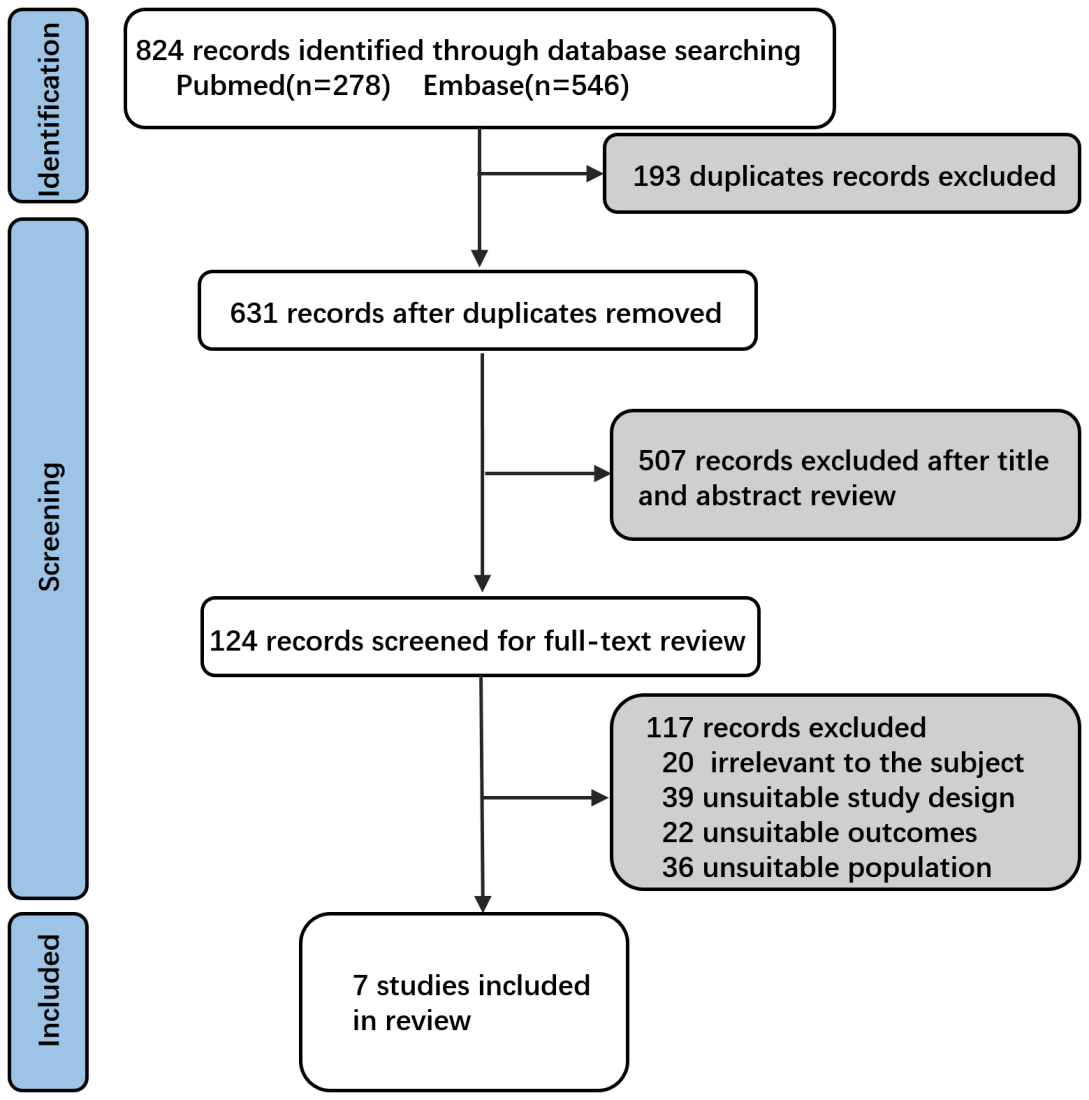
Flowchart of study identification and inclusion.

Based on the search strategy (**Figure 1**), 278 studies were retrieved from PubMed and 546 studies from Embase, totaling 824 studies. A total of 193 duplicates were excluded. Following an initial screening of titles and abstracts, 507 studies were deemed irrelevant and excluded. After applying the selection criteria through a full-text review, an additional 117 studies were excluded (**Supplementary Table 1**). Ultimately, seven studies were included in this meta-analysis (**Figure 1** and **Table 1**) (21–24, 26–28). These 7 studies comprised 4 RCTs (21–24) and 3 observational study (26–28), reporting data on 3987 immunized and 3360 non-immunized preterm infants.

**Table 1:** Characteristics of included studies.

Author	Year	Settings	Design	Observe time *	Gestational age	Total sample	Nirsevimab	Control	Dosage	Primary outcome in original study
Domachowske et al. (21)	2018	RCT study NCT02290340	MC, 10 site, 3 countries	151 days	32 + 0-34 + 6	46	30	16	50mg	medically attended LRTI
Griffin et al. (22)	2020	RCT study NCT02878330	MC, 164 sites, 23 countries	150 days	29 + 0-34 + 6	1453	969	484	50mg	Medically attended RSV associated LRTI
Hammitt et al. (23)	2022	RCT study NCT03979313	MC, 150 sites, 20 countries	150 days	35 + 0-36 + 6	208	132	76	50mg(<5kg) 100mg (≥5kg)	Medically attended RSV associated LRTI
Drysdale et al. (24)	2023	RCT study NCT05437510	MC, 235 sites, 3 countries	during the 22-23 RSV season	29 + 0-36 + 6	1108	567	541	50mg(<5kg) 100mg (≥5kg)	Hospitalization for RSV associated LRTI
Lopez-Lacort et al. (26)	2024	Observational study	MC, Spain 57 primary care PC centers	during 23-24 RSV season	<37	18	16	2	50mg(<5kg) 100mg (≥5kg)	Medically attended RSV-LRTI in outpatient
Jabagi et al. (27)	2025	Observational study	MC, population based in French	September 15, 2023 to January 31, 2024	<37	4470	2235	2235	50mg(<5kg) 100mg (≥5kg)	Hospitalization for RSV associated LRTI
Rius-Peris et al. (28)	2025	Observational study	MC, 20 Spanish hospitals	during 23-24 RSV season	<37	44	38	6	50mg(<5kg) 100mg (≥5kg)	Hospitalization for RSV-associated bronchiolitis

*Observe time after immunization of nirsevimab.

MC, Multi-center; RCT, randomized controlled trial; LRTI, lower respiratory tract infection.

In two out of seven studies, participants immunized with nirsevimab received a single intramuscular injection of 50 mg of nirsevimab (21, 22). In the other five studies, participants received a single intramuscular injection of nirsevimab, with a dose of 50 mg for those weighing less than 5 kg and 100 mg for those weighing 5 kg or more (23, 24, 26–28). In four of the seven studies, the control group received a placebo (21–24), while in the remaining studies, the control group received no intervention (26–28). The primary outcomes were observed over 151 or 150 days post-dose in three of the seven studies (21–23), and during the RSV season in the other four studies (24, 26–28). Among the seven included studies, one focused exclusively on preterm infants (22), while the other four included both preterm and full-term infants, with the majority being full-term infants (21, 23, 24, 26–28).

### 3.2 Risk of bias evaluation

This meta-analysis was evaluated for risk of bias using the Cochrane RoB2 Tool by two independent investigators, as previously mentioned (38). A summary of the findings from the risk of bias assessment is presented in **Table 2**. According to the RoB2 Tool, with the exception of three observational study, the overall risk of bias was low across several domains: randomization process, deviations from the intended interventions, missing data, outcome measurement, and selection of reported results.

**Table 2:** Risk of bias using RoB2 tool.

Author	Year	Randomization process generated	Deviations from the intended intervention	Missing data	Measurement of the outcome	Selection of the reported results	Overall
Domachowske et al. (21)	2018	Low	Low	Low	Low	Low	Low
Griffin et al. (22)	2020	Low	Low	Low	Low	Low	Low
Hammitt et al. (23)	2022	Low	Low	Low	Low	Low	Low
Drysdale et al. (24)	2023	Low	Low	Low	Low	Low	Low
Lopez-Lacort et al. (26)	2024	Some concerns	Some concerns	Low	Low	Low	Some concerns
Jabagi et al. (27)	2025	Some concerns	Some concerns	Low	Low	Low	Some concerns
Rius-Peris et al. (28)	2025	Some concerns	Some concerns	Low	Low	Low	Some concerns

### 3.3 Meta-analysis of main outcomes

**Figure 2:**
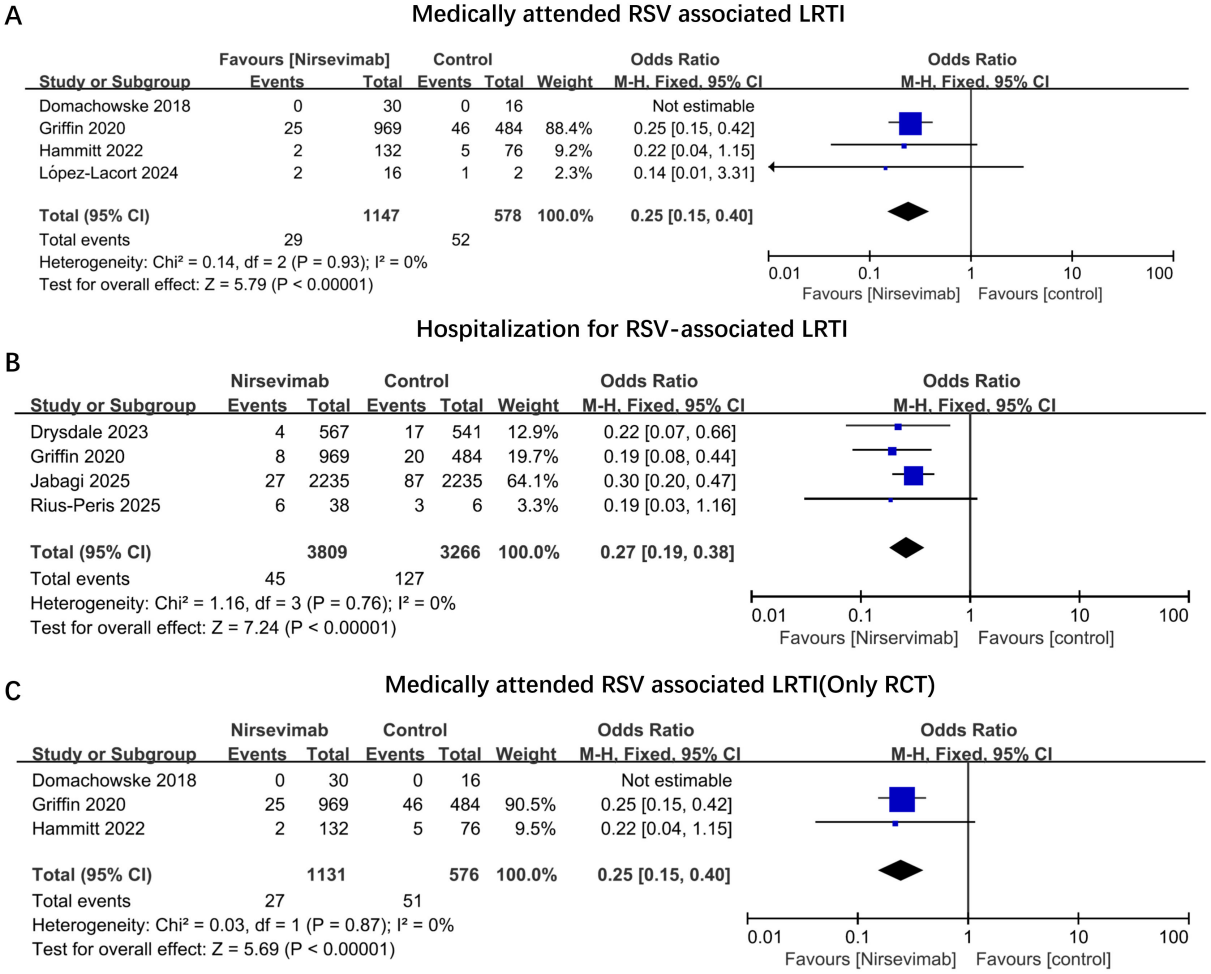
Odds ratios of main outcomes comparing immunized nirsevimab versus non-immunized preterm infants. Forest plots illustrating the odds ratios of **(A)** Medically Attended RSV-Associated LRTIs; **(B)** Hospitalization for RSV-Associated LRTIs; **(C)** Medically Attended RSV-Associated LRTIs (Only in RCT Studies). The vertical ticks within the blue boxes and the horizontal lines represent the mean effect and 95% confidence interval for each study. The black diamond at the bottom indicates the cumulative effect with 95% confidence intervals.

Among the seven studies reviewed, four reported medically attended RSV-associated LRTIs (21–23, 26). Four out of the seven studies documented hospitalizations due to RSV-associated LRTIs (22, 24, 27, 28). No studies provided clear data on the rates and durations of intensive care unit (ICU) admissions, the use of supplemental oxygen, or the application of mechanical ventilation (MV). For medically attended RSV-associated LRTIs, four studies encompassed 1,147 infants in the nirsevimab group and 578 infants in the control group. Immunization with nirsevimab was associated with a 75% reduction in the odds of medically attended RSV-associated LRTIs (pooled odds ratio [OR] 0.25; 95% confidence interval [CI] 0.15, 0.40, P<0.0001, I^2^ = 0%) (**Figure 2A**). Concerning hospitalizations for RSV-associated LRTIs, four studies involving 3809 infants immunized with nirsevimab and 3266 non-immunized infants were evaluated, with a pooled OR of 0.27 (95% CI 0.19, 0.38), P<0.0001, I^2^ = 0% (**Figure 2B**). Additionally, we conducted a pooled analysis of medically attended RSV-associated LRTIs exclusively among RCTs. After excluding the observational study, the pooled OR (95% CI) remained at 0.25 (0.15, 0.40), with an overall effect P<0.0001, I^2^ = 0% (**Figure 2C**). As shown in **Figure 2**, all of outcomes had low heterogeneity. In addition, owing to the limited outcome data extracted from the original studies, subgroup analyses (e.g., based on different gestational ages or birth weights) could not be conducted in the present study.

### 3.4 Sensitivity analysis and publication bias

Sensitivity analysis was conducted employing the leave-one-out method. The resulting pooled estimates were presented in **Supplementary Figure 1**. The removal of single studies did not affect either estimates or heterogeneity of medically attended RSV-associated LRTIs, hospitalizations for RSV-associated LRTIs, and medically attended RSV-associated LRTIs(Only RCT studies). As for publication bias, this analysis was assessed through the visual analysis of the asymmetry of funnel plots. These results were presented in **Supplementary Figure 2**, and all of estimates were scattered and relatively symmetrical suggesting that there was no obvious publication bias.

## 4 Discussion

In recent years, the survival rates of preterm infants have increased, requiring comprehensive management of their long-term multi-system outcomes, particularly the respiratory system (39–41). Preterm infants are at a substantially higher risk of respiratory tract infections compared to full-term infants (42–45). Nirsevimab, as a monoclonal antibody for preventing RSV infection, existing studies suggest it has a good effect (21–28). Although many studies have included preterm infants, up to now, there are no extensive literature reviews and meta-analyses specifically for this population. This study fills this gap. A total of 7 studies were included in this research, among which 4 were RCTs and 3 were observational study (21–24, 26–28). The results indicated the significant benefits of nirsevimab in preventing medically attended RSV-related LRTIs and hospitalizations. This study is the first systematic evaluation of nirsevimab’s role in preventing RSV in preterm infants. This finding offers a valuable preventive strategy for RSV in the preterm population.

Based on these favorable findings, a reduction in RSV-related LRTIs can lead to a decrease in both outpatient visits and hospitalization rates. Once preterm infants are infected with RSV, costs associated with outpatient visits for pathogen confirmation and infection laboratory testing arise. In severe cases, upon hospital admission, additional costs related to treatments such as oxygen therapy, mechanical ventilation, and medications are incurred. Nirsevimab reduces the number of medically attended RSV-related LRTIs and hospitalizations, which directly decreases these expenses. However, the cost-effectiveness of nirsevimab should also be evaluated across different clinical settings. This may result in a varied effectiveness profile of nirsevimab. For instance, in regions where co-infections are prevalent but income levels are low, interactions between co-infections and RSV could influence the cost-effectiveness outcomes of nirsevimab immunization, a factor that may not be as well-controlled in RCTs. In such situations, while medically attended RSV-associated infections or RSV-related hospitalizations may decrease, infection or hospitalization rates associated with other pathogens may increase. Therefore, when assessing the impact of nirsevimab, real-world data from diverse settings should also be considered.

Given that nirsevimab was introduced relatively recently, its large-scale application commenced during the 2023-2024 RSV season (25, 46, 47). In the course of reviewing the literature, numerous studies did incorporate preterm infants; however, the outcomes for this specific subgroup were not reported independently in the research findings (47–49). Consequently, this limitation constrained the number of studies we could include and subjected to a comprehensive analysis. In other systematic review and meta-analysis related to nirsevimab, we identified three studies (34–36). One study, conducted before the 2023-2024 epidemic season, evaluated the effects of different monoclonal antibodies on RSV infection (34). Another study, which included only two trials, focused on the safety and efficacy of nirsevimab in infants (36). The third study, completed in May 2024, encompassed the majority of the most recent research, examining the impact of nirsevimab on the hospitalization rates of infants with RSV (35). In comparison with the aforementioned meta-analysis, our study was predominantly focused on preterm infants. The results from each original study and the meta-analysis were consistently favorable, demonstrating a significant effect of nirsevimab. To enhance our analysis, we conducted an integrated analysis of four RCTs (**Figure 2C**) (21–24), showing a positive effect in preventing RSV-related LRTIs. However, all of these four RCTs were industry-funded. The potential sponsor bias should also be noted and excluded in future studies.

Before making recommendations on the current implementation, it is imperative to give significant consideration to the safety of nirsevimab. However, our study did not evaluate safety due to limitations in the original research. In original studies, only one article provided isolated safety data. In other studies, the safety data were combined with full-term infants and cannot be distinguished (22). Consequently, we were unable to perform a comprehensive safety analysis of nirsevimab in preterm infants. In the sole study focusing on preterm infants, the researchers observed that the incidence of adverse reactions did not significantly differ from that of the control group (22). Additionally, the study by Domachowske et al. specifically addressed the safety of vaccination in this cohort and concluded that the safety profile of nirsevimab was similar to that of palivizumab (33).

Our study encompassed seven research articles, a number that remains relatively modest. During the literature screening process, numerous articles incorporated preterm groups. Regrettably, it was not feasible to isolate the outcomes of nirsevimab for preterm infants from the original literature, which significantly constrained the number of studies included in our analysis. Consequently, we eagerly anticipate future research endeavors that will specifically focus on this unique population. Most of the original studies in this research were from high-income countries, with limited data from low- and middle-income countries. However, the condition is more severe in low- and middle-income countries. We expect more future studies to focus on these regions (9, 50, 51). In China, nirsevimab vaccination has commenced for the 2024-2025 RSV epidemic season. We eagerly await the results following the conclusion of the epidemic season. Additionally, our research primarily focused on medically attended RSV-associated LRTIs and hospitalizations due to RSV-associated LRTIs. However, there is a clinical need for data on overall LRTIs and hospitalizations for any cause, which could serve as secondary outcomes. This aligns better with actual clinical requirements. Indeed, our center is currently conducting an observational study on the immunization of nirsevimab in preterm infants, and we are hopeful that our findings will contribute valuable insights to this field.

This study acknowledges several limitations. Firstly, the sample sizes of certain studies were relatively small, which may limit the generalizability of the findings. Secondly, due to limited data in original studies, detailed analyses based on safety of nirsevimab, different gestational ages, and clinical outcomes (such as duration of oxygen therapy, length of hospital stay, hospitalization costs, mechanical ventilation duration) were not performed in present study. Thirdly, the current included studies mainly come from high-income countries, and there is a lack of data from low-income countries. In addition, considering the influence of RSV on the future respiratory system of preterm infants, nirsevimab preventive effect on long-term prognosis was also limitation of present study. Fourthly, the present study was conducted during the 2024-2025 RSV seasons. This indicates that the majority of ongoing clinical trials on nirsevimab during this RSV season were incomplete and therefore not included. All of these findings may contribute to future meta-analyses and provide more robust evidence for the application of nirsevimab. Lastly, different RSV subtypes and different dosing strategies were also not examined in present study. All of these limitations should be the potential direction for future studies.

## 5 Conclusion

Nirsevimab substantially mitigates the risk of RSV infection and hospitalization among preterm infants, offering a more convenient and efficacious preventive strategy for high-risk preterm infants against RSV, thereby demonstrating extensive potential for clinical utilization. However, due to the limited data and fully follow-up data from original studies, when it comes to real clinical application or guideline suggestion, the following points need to be properly addressed, including the timing selection, the cost-effectiveness, and regional differences.

## Data Availability

The original contributions presented in the study are included in the article/**Supplementary Material**. Further inquiries can be directed to the corresponding author.

## Supplementary material

Supplementary Figure 1 Sensitivity analysis of each outcomes. Forest plot for sensitivity analysis on **(A)** Medically Attended RSV-Associated LRTIs; **(B)** Hospitalization for RSV-Associated LRTIs; **(C)** Medically Attended RSV-Associated LRTIs (Only in RCT Studies). Sensitivity analysis was performed by excluding one study at a time and reporting corresponding pooled estimates with heterogeneity estimates.

Supplementary Figure 2 Analysis of publication bias by visual analysis of the asymmetry of funnel plots. Funnel plots for prevalence data on the **(A)** Medically Attended RSV-Associated LRTIs; **(B)** Hospitalization for RSV-Associated LRTIs; **(C)** Medically Attended RSV-Associated LRTIs (Only in RCT Studies).
