# Supplementary figures and images for: Effectiveness of nirsevimab immunization against RSV infection in preterm infants: a systematic review and meta-analysis
Source: Front Immunol. 2025 Apr 17;16:1581970. doi: 10.3389/fimmu.2025.1581970 (PMC12043638; doi:10.3389/fimmu.2025.1581970)

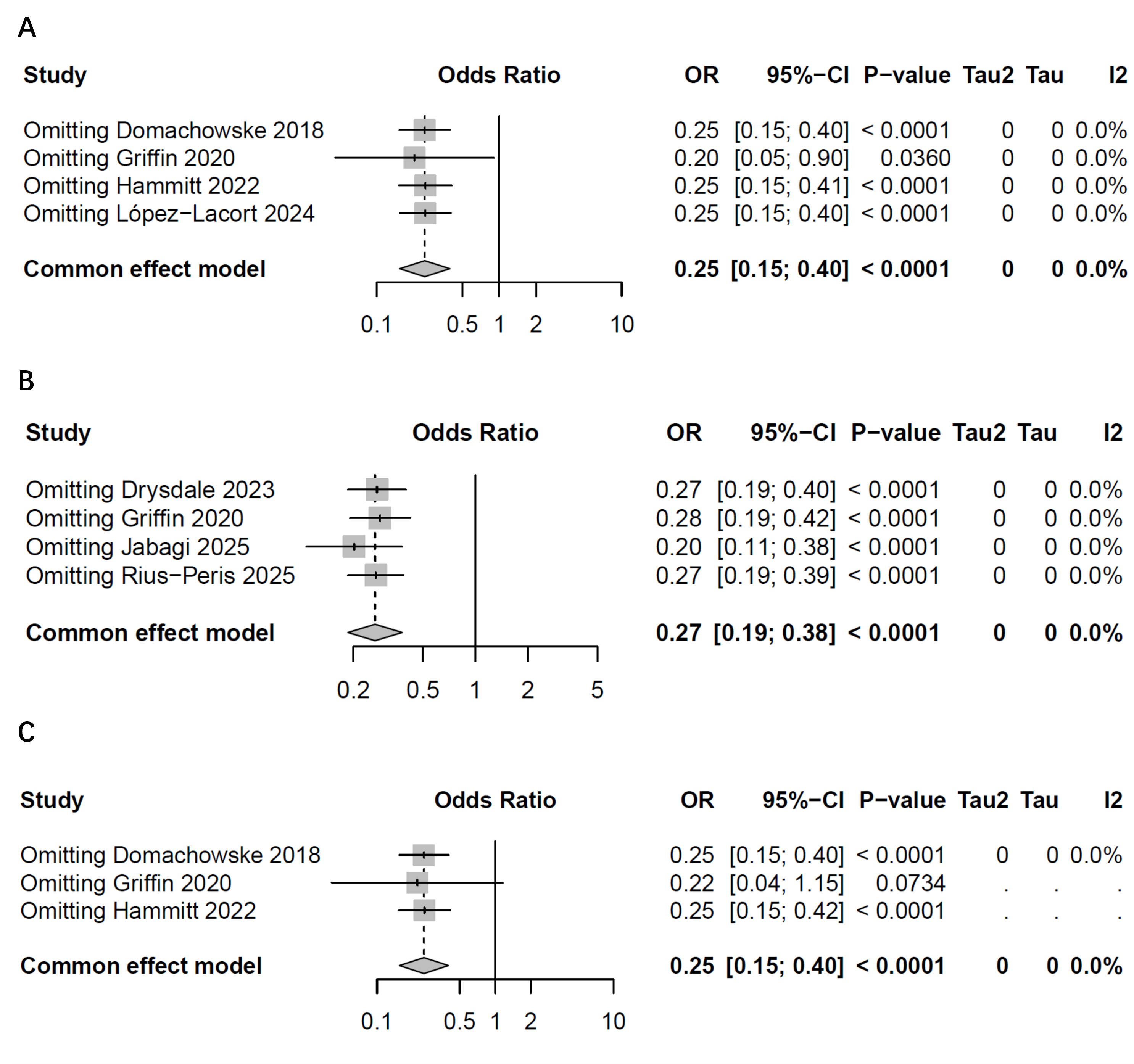

Supplement: Supplementary Figure 1 — Sensitivity analysis of each outcomes. Forest plot for sensitivity analysis on (A) Medically Attended RSV-Associated LRTIs; (B) Hospitalization for RSV-Associated LRTIs; (C) Medically Attended RSV-Associated LRTIs (Only in RCT Studies). Sensitivity analysis was performed by excluding one study at a time and reporting corresponding pooled estimates with heterogeneity estimates. [file Image1.tif]

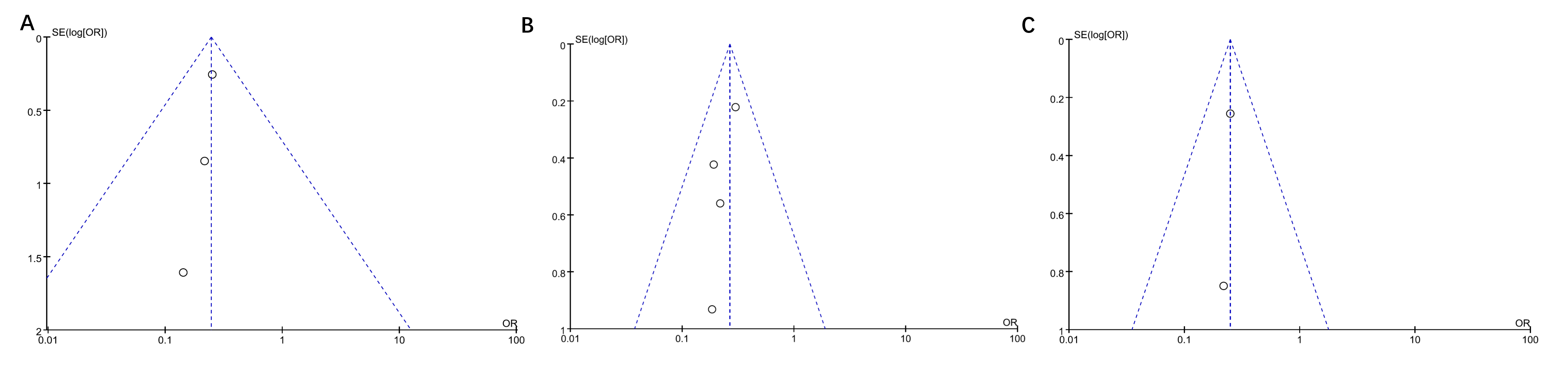

Supplement: Supplementary Figure 2 — Analysis of publication bias by visual analysis of the asymmetry of funnel plots. Funnel plots for prevalence data on the (A) Medically Attended RSV-Associated LRTIs; (B) Hospitalization for RSV-Associated LRTIs; (C) Medically Attended RSV-Associated LRTIs (Only in RCT Studies). [file Image2.tif]
